# Supplementary material for: High-Resolution Taxonomic Characterization Reveals Novel Human Microbial Strains with Potential as Risk Factors and Probiotics for Prediabetes and Type 2 Diabetes
Source: Microorganisms. 2023 Mar 15;11(3):758. doi: 10.3390/microorganisms11030758 (PMC10051885; doi:10.3390/microorganisms11030758)
Supplement: Supplementary file 1 [file microorganisms-11-00758-s001.zip › Hendricks_etal_Supplementary_Microorganisms-2230320.pdf]

Supplementary Materials
Results

Differentially abundant taxa
When comparing type 2 diabetes to healthy patients, relative abundance differed significantly for a total of 21 OTUs. Five taxa representing five genera, *Absiella*, *Burkholderiales*, *Escherichia*, *Negativibacillus*, and *Odoribacter*, were enriched in fecal samples of type 2 diabetes patients (Table 6 and Figure 3). Of these, two were classified to the strain level (*Escherichia coli* O1:H42 and *Odoribacter laneus* YIT 12061). The other 15 OTUs were enriched in the fecal samples of healthy patients, representing ten genera (*Anaerostipes*, *Bacteroides*, *Catenibacterium*, *Escherichia*, *Intestinibacter*, *Megamonas*, *Paeniclostridium*, *Prevotella*, *Romboutsia*, and *Selenomonas* genera), one *Mollicutes* class, and two families (*Hungateiclostridiaceae* and *Peptostreptococcaceae*). Of these 15 OTUs, one was identified at the species level (*Romboutsia timonensis*) and four were identified at strain level (*Bacteroides coprophilus* DSM 18228 = JCM 13818, *Bacteroides stercoris* ATCC 43183, *Catenibacterium mitsuokai* DSM 15897, *Escherichia coli* O28:H27, and *Prevotella copri* DSM 18205).

The comparison between prediabetes and healthy showed that the relative abundance differed for a total of 21 OTUs. Of these, six were more abundant in fecal samples of prediabetes patients (Table 6 and Figure 3), including strains *Odoribacter laneus* YIT 12061, *[Bacteroides] pectinophilus* ATCC 43243, *Escherichia coli* O25b:H4-ST131, and *Erysipelatoclostridium ramosum* DSM 1402, and one OTU from the family *Catabacteriaceae* and one from the species *Streptococcus thermophilus*. The other fifteen OTUs were enriched in healthy individuals, including two in the genera *Megasphaera* and *Selenomonas*. The OTUs identified to species were *Lactobacillus animalis*, *Lactobacillus murinus*, *Bifidobacterium pseudocatenulatum*, *Megasphaera elsdenii* and *Acidaminococcus intestini*. The remaining eight were identified to strain (*Bifidobacterium adolescentis* ATCC 15703, *Slackia piriformis* YIT 12062, *Acidaminococcus intestine* RyC-MR95, *Escherichia coli* O1:H42, *Escherichia coli* O128:H27, *Catenibacterium mitsuokai* DSM 15897, *Bifidobacterium pseudocatenulatum* DSM 20438, and *Bacteroides coprophilus* DSM 18228 = JCM 13818).

Our study revealed a total of 10 OTUs that were significantly different in the relative abundance between prediabetes/healthy and healthy individuals. Of these, four were enriched in prediabetes/healthy patients, including *Odoribacter laneus* YIT 12061, *Bacteroides coprocola* DSM 17136, and two OTUs from the genus *Streptococcus* including one identified to species, *Streptococcus salivarius*. In addition, six OTUs were enriched in healthy individuals as compared to prediabetic/healthy individuals with one OTU in the family of *Flavobacteriaceae*, and two identified to species, *Lactobacillus animalis* and *Klebsiella pneumoniae*. The others were identified to strain and included *Escherichia coli* O25b:H4-ST131, *Escherichia coli* O25b:H4, *Bacteroides coprophilus* DSM 18228 = JCM 13818.

The microbial composition differed between prediabetes/healthy and individuals with prediabetes. Here, we observed that eight OTUs were enriched in prediabetic and ten were enriched in prediabetes/healthy individuals. The eight more abundant OTUs in prediabetes were found in the families of *Hungateiclostridiaceae* and *Flavobacteriaceae*. One was identified to the species *Klebsiella pneumoniae* and two from the species *Lactococcus lactis*, one of which

was identified to the strain *Lactococcus lactis* subsp. lactis Il1403. The additional OTUs were identified to strain including *[Bacteroides] pectinophilus* ATCC 43243 and two *Escherichia coli* strains, *Escherichia coli* O25b:H4 and *Escherichia coli* O25b:H4-ST131. As for the OTUs enriched in prediabetes/healthy - two OTUs were identified to the genus, *Selenomonas* and *Megasphaera*, and three to the species *Acidaminococcus intestini*, *Bifidobacterium pseudocatenulatum*, and *Megasphaera elsdenii*. Five strains were identified (*Slackia piriformis* YIT 12062, *Bifidobacterium pseudocatenulatum* DSM 20438, *Acidaminococcus intestine* RyC-MR95, *Bacteroides coprocola* DSM 17136, and *Escherichia coli* O128:H27).

Interesting and significant differences were also noted upon comparing prediabetes/healthy to type 2 diabetes. The individuals with type 2 diabetes had more abundance in the following ten OTUs: *Flavobacteriaceae* (Family); *Lactobacillus johnsonii*, *Lactobacillus animalis*, *Klebsiella pneumoniae*, *Alistipes sp. 5CPEGH6*, *Bacteroides vulgatus,* and *Escherichia coli* (species); and *Bacteroides vulgatus* ATCC 8482, *Escherichia coli* O25b:H4 and *Escherichia coli* O25b:H4-ST131 (strain). Twelve OTUs were enriched in prediabetic/healthy individuals. These OTUs were classified as follows: one to class (*Mollicutes*), one to family (*Peptostreptococcaceae*), five to genus (*Streptococcus*, *Catenibacterium*, *Megamonas*, and *Selenomonas*), one to species (*Streptococcus salivarius*) and five to strain (*Catenibacterium mitsuokai DSM* 15897, *Prevotella copri* DSM 18205, *Escherichia coli* O128:H27, *Bacteroides stercoris* ATCC 43183 and *Bacteroides coprocola* DSM 17136).

Finally, comparison of prediabetes to type 2 diabetes identified 15 OTUs, which were more abundant in type 2 diabetes than in prediabetes. These included *Acidaminococcus intestini* (species) and *Acidaminococcus intestine* RyC-MR95 (strain), *Flavonifractor* (genus) and *Flavonifractor plautii* (species), *Lactobacillus johnsonii* (species), *Lactobacillus animalis* (species) and *Lactobacillus murinus* (species), *Bifidobacterium pseudocatenulatum* (species) and *Bifidobacterium pseudocatenulatum* DSM 20438 = JCM 1200 = LMG 10505 (strain), *Megasphaera* (genus) and *Megasphaera elsdenii* (species) as well as the three additional taxa of Lachnoclostridium (genus), *Bacteroides vulgatus* (species), *Escherichia coli* O1:H42 (strain), and *Slackia piriformis* YIT 12062 (strain). Eighteen OTUs were more abundant in prediabetes as compared to type 2 diabetes individuals. These included OTUs belonging to the families of *Catabacteriaceae*, *Hungateiclostridiaceae* and *Peptostreptococcaceae*; genera of *Mollicutes, Anaerostipes, Catenibacterium*, *Clostridium*, *Christensenella*, *Romboutsia*, *Robinsoniella*, *Paeniclostridium*, and *Intestinibacter*; species of *Streptococcus thermophilus*, *Romboutsia timonensis*; and strains of *[Bacteroides] pectinophilus* ATCC 43243, *Prevotella copri* DSM 18205, *Bacteroides stercoris* ATCC 43183, and *Catenibacterium mitsuokai* DSM 15897.

Tables

Table S1. Sample metadata (ID, health status, sex) as well as number of raw reads. The number of counts of Firmicutes and Bacteroidetes and F/B ratios are also given.

Sample ID	Health status	Age, years	Sex	Number of raw reads	Firmicutes, counts	Bacteroidetes, counts	FB Ratio
SG1	Diabetes	57	Female	8133	2548	3751	0.6793
SG2	Diabetes	49	Female	9815	3444	3751	0.9182
SG31	Diabetes	60	Female	30542	14441	4851	2.9769
SG43	Diabetes	63	Male	19664	2928	11777	0.2486
SG44	Diabetes	64	Female	11068	4226	1400	3.0186
SG47	Diabetes	70	Female	12347	2621	6403	0.4093
SG48	Diabetes	58	Male	8943	4462	2014	2.2155
SG5	Diabetes	32	Male	11011	5882	4234	1.3892
SG51	Diabetes	52	Male	14583	6933	2128	3.2580
SG40	Diabetes	45	Male	11818	2296	2578	0.8906
SG10	Healthy	38	Female	13042	7961	2701	2.9474
SG13	Healthy	24	Female	3253	1728	598	2.8896
SG14	Healthy	53	Female	15116	7218	5745	1.2564
SG15	Healthy	29	Female	14926	6983	4105	1.7011
SG17	Healthy	72	Male	13560	5390	5119	1.0529
SG19	Healthy	69	Male	12461	10243	709	14.4471
SG20	Healthy	25	Female	18999	9155	7601	1.2044
SG21	Healthy	26	Male	16507	5225	8700	0.6006
SG22	Healthy	33	Female	12825	7274	2032	3.5797
SG23	Healthy	70	Female	12923	7906	1714	4.6126
SG24	Healthy	67	Female	15963	8306	2669	3.1120
SG25	Healthy	29	Male	15849	9752	3227	3.0220
SG26	Healthy	35	Female	17266	9113	2894	3.1489
SG27	Healthy	42	Male	11085	3374	651	5.1828
SG28	Healthy	37	Female	14256	4603	538	8.5558
SG29	Healthy	25	Male	15553	3594	391	9.1918
SG3	Healthy	49	Female	13048	5149	5775	0.8916
SG30	Healthy	24	Female	23199	17416	2447	7.1173
SG42	Healthy	69	Female	12023	6700	3915	1.7114
SG46	Healthy	41	Male	11932	6099	303	20.1287
SG9	Healthy	21	Male	17680	9937	2032	4.8903
SG18	Prediabetes	79	Female	25866	17398	612	28.4281
SG32	Prediabetes	53	Male	18415	11246	925	12.1578
SG33	Prediabetes	64	Male	18734	11221	3412	3.2887

SG36	Prediabetes	44	Male	21597	11808	7091	1.6652
SG37	Prediabetes	56	Female	18650	10634	844	12.5995
SG39	Prediabetes	66	Female	14855	6929	4761	1.4554
SG49	Prediabetes	54	Female	11236	6560	2902	2.2605
SG8	Prediabetes	62	Female	14339	6971	1134	6.1473
G11	Prediabetes/healthy	54	Male	4	Removed	Removed	Removed
SG16	Prediabetes/healthy	64	Female	18067	11885	1282	9.2707
SG34	Prediabetes/healthy	55	Male	16974	12524	1717	7.2941
SG35	Prediabetes/healthy	61	Female	37935	25907	3015	8.5927
SG38	Prediabetes/healthy	50	Female	34408	18346	4920	3.7289
SG4	Prediabetes/healthy	69	Female	12601	7944	12	662.0000
SG41	Prediabetes/healthy	63	Female	11610	7144	2414	2.9594
SG45	Prediabetes/healthy	69	Female	14546	9685	2058	4.7060
SG6	Prediabetes/healthy	58	Male	15105	7352	2161	3.4021

Table S2. Percentage of each phylum present for each sample.

Sample ID	Firmicutes	Bacteroidetes	Actinobacteria	Proteobacteria	Verrucomicrobia	Tenericutes	Elusimicrobia	Synergistetes	Fusobacteria	Lentisphaerae	Cyanobacteria
SG1	33.34%	49.08%	0.75%	5.72%	10.99%	0.05%	0.00%	0.03%	0.00%	0.04%	0.00%
SG10	66.16%	22.45%	3.00%	1.26%	0.01%	7.05%	0.00%	0.06%	0.00%	0.00%	0.00%
SG13	61.32%	21.22%	12.92%	1.63%	2.91%	0.00%	0.00%	0.00%	0.00%	0.00%	0.00%
SG14	53.49%	42.57%	1.96%	0.27%	1.70%	0.01%	0.00%	0.00%	0.00%	0.00%	0.00%
SG15	52.33%	30.76%	9.60%	3.75%	3.55%	0.01%	0.00%	0.00%	0.00%	0.00%	0.00%
SG16	78.73%	8.49%	8.63%	3.41%	0.73%	0.01%	0.00%	0.00%	0.00%	0.00%	0.00%
SG17	47.86%	45.46%	4.96%	1.28%	0.30%	0.12%	0.00%	0.00%	0.00%	0.00%	0.00%
SG18	89.55%	3.15%	4.69%	2.61%	0.00%	0.00%	0.00%	0.00%	0.00%	0.00%	0.00%
SG19	84.31%	5.84%	9.81%	0.04%	0.00%	0.00%	0.00%	0.00%	0.00%	0.00%	0.00%
SG2	41.90%	45.64%	8.41%	4.00%	0.05%	0.00%	0.00%	0.00%	0.00%	0.00%	0.00%
SG20	48.58%	40.33%	7.53%	3.57%	0.00%	0.00%	0.00%	0.00%	0.00%	0.00%	0.00%
SG21	34.87%	58.06%	5.16%	1.89%	0.02%	0.00%	0.00%	0.00%	0.00%	0.00%	0.00%
SG22	58.19%	16.25%	8.56%	16.90%	0.10%	0.00%	0.00%	0.00%	0.00%	0.00%	0.00%
SG23	61.84%	13.41%	11.83%	11.26%	1.50%	0.00%	0.00%	0.16%	0.00%	0.00%	0.00%
SG24	62.89%	20.21%	3.95%	3.04%	2.16%	7.69%	0.00%	0.00%	0.00%	0.00%	0.03%
SG25	65.61%	21.71%	10.99%	1.53%	0.15%	0.01%	0.00%	0.00%	0.00%	0.00%	0.00%
SG26	63.68%	20.22%	5.34%	4.44%	0.01%	4.75%	1.49%	0.00%	0.00%	0.00%	0.00%
SG27	35.96%	6.94%	55.95%	0.33%	0.35%	0.32%	0.00%	0.15%	0.00%	0.00%	0.00%
SG28	77.66%	9.08%	4.74%	2.21%	6.31%	0.00%	0.00%	0.00%	0.00%	0.00%	0.00%
SG29	47.65%	5.18%	41.63%	5.53%	0.01%	0.00%	0.00%	0.00%	0.00%	0.00%	0.00%
SG3	42.47%	47.63%	4.40%	0.86%	4.64%	0.00%	0.00%	0.00%	0.00%	0.00%	0.00%
SG30	76.12%	10.69%	7.58%	5.53%	0.00%	0.08%	0.00%	0.00%	0.00%	0.00%	0.00%
SG31	64.02%	21.51%	13.03%	1.33%	0.00%	0.00%	0.00%	0.00%	0.11%	0.00%	0.00%
SG32	61.63%	5.07%	25.58%	7.40%	0.01%	0.00%	0.00%	0.00%	0.31%	0.00%	0.00%

SG33	69.98%	21.28%	5.24%	3.12%	0.01%	0.32%	0.00%	0.00%	0.00%	0.01%	0.00%
SG34	80.11%	10.98%	7.13%	1.30%	0.00%	0.14%	0.00%	0.00%	0.32%	0.00%	0.00%
SG35	88.47%	10.30%	0.55%	0.42%	0.09%	0.00%	0.00%	0.02%	0.16%	0.00%	0.00%
SG36	56.24%	33.77%	4.34%	5.63%	0.01%	0.00%	0.00%	0.00%	0.00%	0.00%	0.00%
SG37	72.08%	5.72%	15.68%	4.81%	1.71%	0.00%	0.00%	0.00%	0.01%	0.00%	0.00%
SG38	63.63%	17.07%	17.91%	0.43%	0.95%	0.00%	0.00%	0.00%	0.00%	0.00%	0.00%
SG39	50.14%	34.45%	14.44%	0.97%	0.00%	0.00%	0.00%	0.00%	0.00%	0.00%	0.00%
SG4	64.29%	0.10%	12.24%	13.11%	10.25%	0.01%	0.00%	0.00%	0.00%	0.00%	0.00%
SG40	19.98%	22.44%	32.33%	25.22%	0.02%	0.00%	0.00%	0.00%	0.01%	0.00%	0.00%
SG41	64.72%	21.87%	9.69%	1.95%	0.05%	1.71%	0.00%	0.00%	0.00%	0.00%	0.00%
SG42	58.23%	34.03%	4.69%	2.69%	0.13%	0.21%	0.00%	0.00%	0.00%	0.00%	0.00%
SG43	15.63%	62.85%	2.47%	17.13%	1.93%	0.00%	0.00%	0.00%	0.00%	0.00%	0.00%
SG44	58.22%	19.29%	12.11%	9.97%	0.41%	0.00%	0.00%	0.00%	0.00%	0.00%	0.00%
SG45	71.80%	15.26%	11.15%	0.40%	1.39%	0.00%	0.00%	0.00%	0.00%	0.00%	0.00%
SG46	84.30%	4.19%	10.60%	0.90%	0.01%	0.00%	0.00%	0.00%	0.00%	0.00%	0.00%
SG47	21.60%	52.76%	5.00%	20.63%	0.00%	0.00%	0.00%	0.00%	0.00%	0.00%	0.00%
SG48	56.73%	25.60%	3.59%	14.09%	0.00%	0.00%	0.00%	0.00%	0.00%	0.00%	0.00%
SG49	61.99%	27.42%	4.69%	2.28%	0.28%	3.26%	0.00%	0.05%	0.00%	0.03%	0.00%
SG5	53.66%	38.62%	4.30%	3.42%	0.00%	0.00%	0.00%	0.00%	0.00%	0.00%	0.00%
SG51	53.22%	16.33%	22.16%	8.28%	0.01%	0.00%	0.00%	0.00%	0.00%	0.00%	0.00%
SG6	64.72%	19.02%	5.70%	4.54%	0.01%	0.29%	0.00%	0.48%	0.00%	0.00%	0.00%
SG8	60.83%	9.90%	11.74%	1.55%	13.25%	2.62%	0.00%	0.10%	0.00%	0.00%	0.00%
SG9	62.86%	12.86%	14.97%	9.31%	0.00%	0.00%	0.00%	0.00%	0.00%	0.00%	0.00%
**Mean**	58.80%	23.13%	10.72%	5.15%	1.40%	0.61%	0.03%	0.02%	0.02%	0.00%	0.00%
**Stdev**	16.8516	15.8951	10.3704	5.7577	2.9918	1.7115	0.2171	0.0775	0.0692	0.007	0.0044

Table S3. Number of known, previously published and unclassified taxon at each classification level.

	Total, count	Classified, count	Unclassified, count	Classified, %	Unclassified, %
Kingdom	1	1	0	100.00%	0.00%
Phylum	11	10	1	90.91%	9.09%
Class	29	23	6	79.31%	20.69%
Order	62	38	24	61.29%	38.71%
Family	114	83	31	72.81%	27.19%
Genus	312	213	99	68.27%	31.73%
Species	602	308	294	51.16%	48.84%
Strain	876	314	562	35.84%	64.16%

Table S4. Fold change (log 2) of differentially abundant phyla from all health category comparisons.

Comparison	Phylum	log2FoldChange	*p* value	*p* adj
Diabetic vs Healthy	Firmicutes	1.04846	0.00037	0.00205
Diabetic vs Healthy	Proteobacteria	-1.21321	0.01270	0.04656
Diabetic vs Healthy	Tenericutes	7.94309	0.00010	0.00109
Diabetic vs Prediabetic	Tenericutes	7.72461	0.00176	0.01940
Diabetic vs Prediabetic/healthy	Firmicutes	1.49471	0.00004	0.00047
Diabetic vs Prediabetic/healthy	Proteobacteria	-1.50739	0.01251	0.03440
Diabetic vs Prediabetic/healthy	Tenericutes	7.05698	0.00465	0.01705

Table S5. Fold change (log 2) of differentially abundant genera from all health category comparisons.

Comparison	Genus	log2FoldChange	*p* value	*p* adj
Diabetic vs Healthy	Paeniclostridium	4.047795632	0.000102123	0.002536067
Diabetic vs Healthy	Romboutsia	5.147650147	1.20E-05	0.000357121
Diabetic vs Healthy	Catenibacterium	18.87542438	1.08E-17	8.05E-16
Diabetic vs Healthy	Megamonas	17.24896681	9.80E-06	0.000357121
Diabetic vs Healthy	Selenomonas	14.3501962	0.000246222	0.005241015
Prediabetic vs Healthy	[Bacteroides]	-6.047341321	0.000239683	0.023968308
Prediabetic vs Healthy	Catenibacterium	8.150274133	0.00038536	0.02890197
Prediabetic vs Healthy	Selenomonas	26.687075	2.05E-10	3.08E-08
Prediabetic vs Healthy	Megasphaera	24.511783	3.83E-11	1.15E-08
Prediabetic/healthy vs Healthy	Streptococcus	-4.509319123	7.75E-07	0.000116303
Diabetic vs Prediabetic	Bacteroides	-1.977177828	0.00365156	0.048312945
Diabetic vs Prediabetic	Robinsoniella	2.922793967	0.001912785	0.0328999
Diabetic vs Prediabetic	Paeniclostridium	4.74811303	0.000194148	0.00465339
Diabetic vs Prediabetic	Romboutsia	6.101448124	1.06E-05	0.000515758
Diabetic vs Prediabetic	Flavonifractor	-4.110793115	0.000216437	0.00465339
Diabetic vs Prediabetic	[Bacteroides]	5.772499397	0.002295153	0.035887848
Diabetic vs Prediabetic	Catenibacterium	10.72515025	6.83E-05	0.001957303
Diabetic vs Prediabetic	Acidaminococcus	-10.58368285	0.002834945	0.040634214
Diabetic vs Prediabetic	Megasphaera	-27.83945504	4.45E-11	3.82E-09
Prediabetic vs Prediabetic/healthy	Lactococcus	-6.195803393	7.48E-05	0.005610322
Prediabetic vs Prediabetic/healthy	Selenomonas	34.66960922	4.66E-12	1.40E-09
Prediabetic vs Prediabetic/healthy	Megasphaera	23.29462624	1.63E-07	1.63E-05
Diabetic vs Prediabetic/healthy	Bacteroides	-2.658384826	0.000108805	0.0040802
Diabetic vs Prediabetic/healthy	Streptococcus	5.19275434	8.41E-07	8.41E-05
Diabetic vs Prediabetic/healthy	Catenibacterium	12.71845288	2.41E-06	0.00016457
Diabetic vs Prediabetic/healthy	Megamonas	22.32142142	3.29E-06	0.00016457
Diabetic vs Prediabetic/healthy	Selenomonas	22.33273042	3.26E-06	0.00016457
Diabetic vs Prediabetic/healthy	Escherichia	-5.14010038	0.000591056	0.019701869
Diabetic vs Prediabetic/healthy	Klebsiella	-9.581452602	9.12E-05	0.003908672

Table S6. See Excel spread sheet: Table S6
Table S7. See Excel spread sheet: Table S7

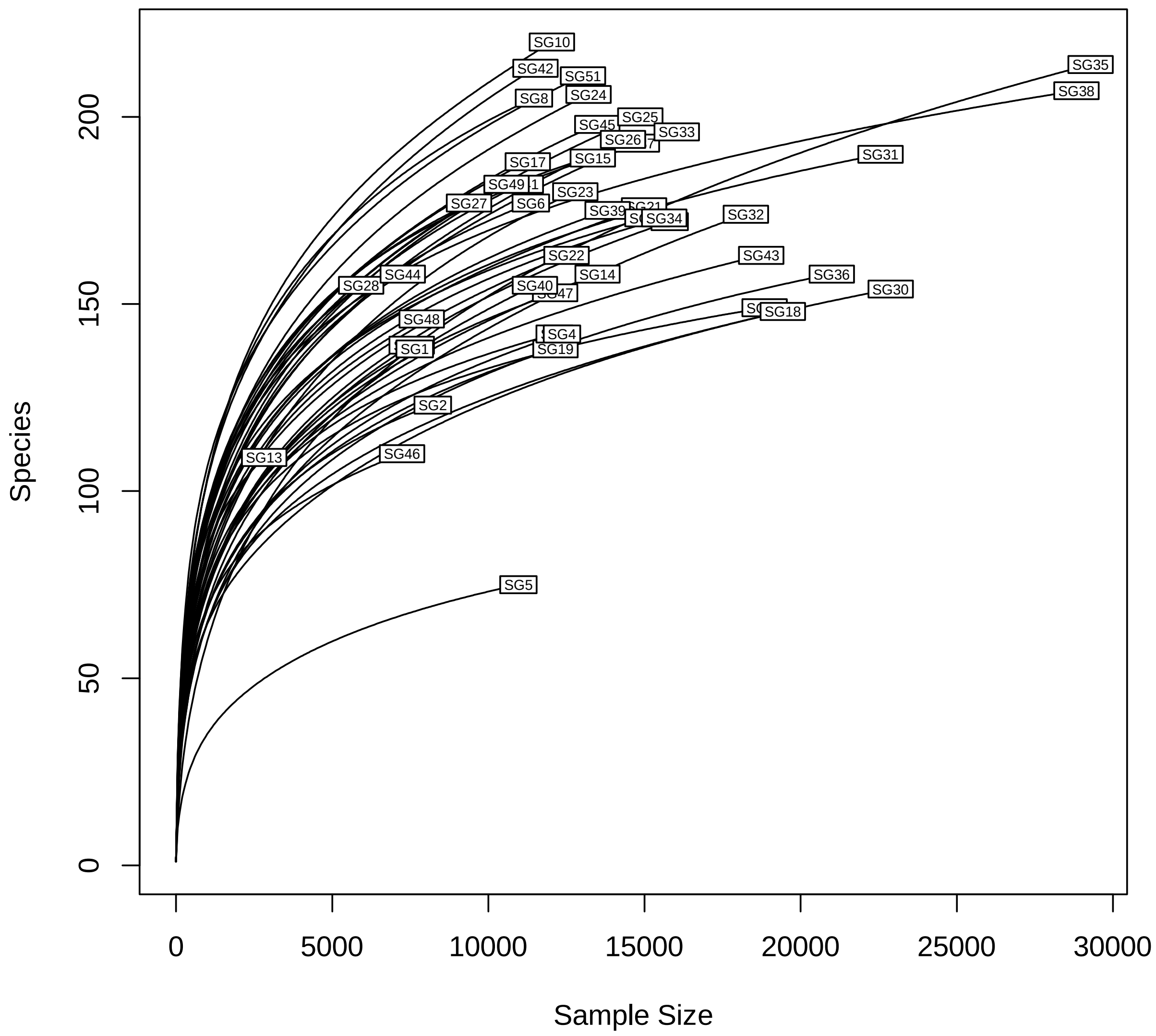

Figure S1. Rarefaction curves showing observed species richness in all samples.

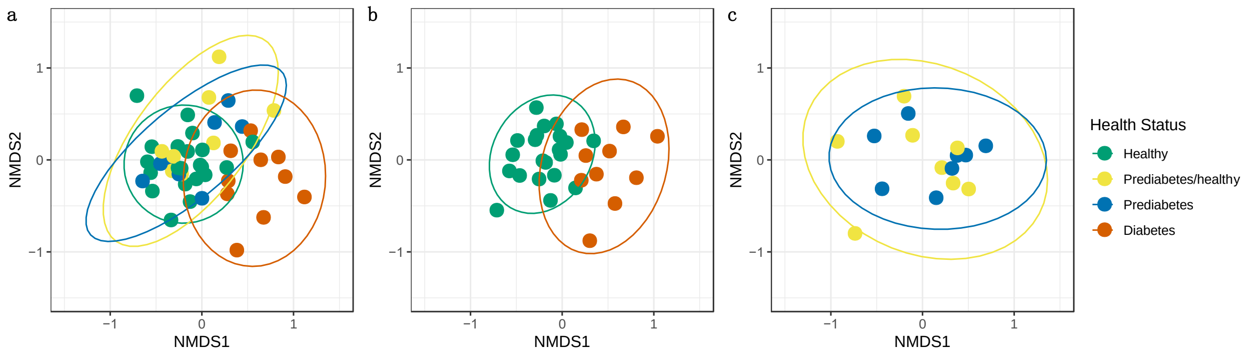

Figure S2. Microbial beta diversity using discriminatory taxa for fecal samples from all 4 health status categories. a) Dimensional reduction of the Bray-Curtis distance between all microbiome samples, using NMDS ordination method. b) NMDS including only samples from prediabetics and those healthy but diagnosed with prediabetes. c) NMDS including only samples from healthy controls and type 2 diabetics. Data points are colored according to health status.
